# Editors’ Note

**DOI:** 10.5195/ijt.2015.6172

**Published:** 2015-07-29

**Authors:** ELLEN R. COHN, JANA CASON

## Abstract

The Spring 2015 issue of the *International Journal of Telerehabilitation* (IJT) contains original research that analyzes provider perspectives on the use of telepractice to serve the families of children who are deaf or hard of hearing. Two case studies examine the utility of administering Facial Affect Training through telepractice to individuals with chronic traumatic brain injury. An article in IJT’s policy section describes the process for the formulation of the World Federation of Occupational Therapists (WFOT) international telehealth position statement for occupational therapy. The World Federation of Occupational Therapists (WFOT) consists of 84 member organizations representing over 420,000 occupational therapists internationally (WFOT, 2014). The Spring 2015 issue also contains announcements of upcoming conferences. As of May 2015, the International Journal of Telerehabilitation (IJT) is live on PubMed Central: http://www.ncbi.nlm.nih.gov/pmc/journals/2411/. Articles from all past issues were indexed, as will be the current and future issues.

The Spring 2015 issue of the *International Journal of Telerehabilitation* (IJT) contains a mix of research, clinical case studies, an article that documents the genesis of the World Federation of Occupational Therapists’ (WFOT) International Telehealth Position Statement for Occupational Therapy, and announcements of major association sponsored meetings.

In this issue, Behl and Kahn shared provider perspectives on the provision of telepractice to families of children who are deaf or hard of hearing. Williamson and Isaki presented two case studies on the novel use of Facial Affect Training through telepractice to individuals with chronic traumatic brain injury. Jacobs et al. reported upon the process for the formulation of the World Federation of Occupational Therapists (WFOT) International Telehealth Position Statement for Occupational Therapy. The World Federation of Occupational Therapists (WFOT) consists of 84 member organizations representing over 420,000 occupational therapists internationally (WFOT, 2014). This policy statement was previously published in IJT.

## PUBMED CENTRAL INDEXING

In May 2015, the International Journal of Telerehabilitation (IJT) became live on PubMed Central: http://www.ncbi.nlm.nih.gov/pmc/journals/2411/. Articles from all past issues are now indexed, as will be the current and future issues.

## NEW EDITORIAL PERSONNEL

We welcome two new additions to the IJT editorial team: Sections Editor William E. Janes, OTD, MSCI, OTR/L and Layout Editor Erin M. Clephas and thank them for their service.

## CALL FOR SUBMISSIONS

The next volume of the International Journal of Telerehabilitation will be published in late Fall, 2015. We cordially invite your submissions by September 1, 2015. IJT accepts original research, case studies, viewpoints, technology reviews, book reviews, and country reports that detail the current status of telerehabilitation. Our peer reviewers constitute a multi-disciplinary group, and include researchers and clinicians from each of the major rehabilitation disciplines, rehabilitation engineers, health information managers, information technologists, and others. We welcome new peer-reviewers and invite guest editors with ideas for special, thematically focused issues. The IJT publication team is agile and can add additional issues as warranted to ensure currency. Please contact Editor Ellen Cohn, PhD (ecohn@pitt.edu) or Senior Associate Editor Jana Cason (jcason@spalding.edu) if you are interested in contributing to a future issue.

Respectfully,

Ellen R. Cohn, PhD, CCC-SLP

IJT Editor

Jana Cason, DHS, OTR/L, FAOTA

Issue Co-Editor and IJT Senior Associate Editor

